# Neuronal representation of duration discrimination in the monkey striatum

**DOI:** 10.14814/phy2.12283

**Published:** 2015-02-12

**Authors:** Atsushi Chiba, Ken-ichi Oshio, Masahiko Inase

**Affiliations:** Department of Physiology, Kinki University Faculty of MedicineOsaka-Sayama, Japan

**Keywords:** Duration discrimination, monkey, neuronal activity, striatum, time perception

## Abstract

Functional imaging and lesion studies in humans and animals suggest that the basal ganglia are crucial for temporal information processing. To elucidate neuronal mechanisms of interval timing in the basal ganglia, we recorded single-unit activity from the striatum of two monkeys while they performed a visual duration discrimination task. In the task, blue and red cues of different durations (0.2–2.0 sec) were successively presented. Each of the two cues was followed by a 1.0 sec delay period. The animals were instructed to choose the longer presented colored stimulus after the second delay period. A total of 498 phasically active neurons were recorded from the striatum, and 269 neurons were defined as task related. Two types of neuronal activity were distinguished during the delay periods. First, the activity gradually changed depending on the duration of the cue presented just before. This activity may represent the signal duration for later comparison between two cue durations. The activity during the second cue period also represented duration of the first cue. Second, the activity changed differently depending on whether the first or second cue was presented longer. This activity may represent discrimination results after the comparison between the two cue durations. These findings support the assumption that striatal neurons represent timing information of sensory signals for duration discrimination.

## Introduction

Interval timing is one of the fundamental constituents of sensory and motor control processes, and pervades many aspects of our lives. Pathophysiological studies of neurological and psychiatric conditions such as Parkinson's disease, schizophrenia, and attention deficit hyperactivity disorder have revealed a key role for the cerebral cortex and basal ganglia in time perception (Allman and Meck 2012). Based on the Striatal Beat-Frequency model (Matell and Meck 2004), striatal neurons monitor oscillatory activity in the cerebral cortex and act as a coincidence detector. The cerebellum also plays an important role in precise timing (Ivry and Spencer 2004). A unified timing model was also proposed in which both the cortico-basal ganglia and olivocerebellar networks are activated and play complementary roles in time perception (Teki et al. 2012). In this model interval timing involves beat-based striatal activation followed by absolute cerebellar timing mechanisms. Temporal information should be represented by neuronal activity within these neural networks in some forms. Recording of neuronal activity from these brain regions in behaving animals is indispensable for elucidating the information processing through these networks and for understanding neuronal mechanisms of time perception.

Ramping neuronal activity, which is ramp-like increases in activity that peak at the end of a timed interval, has been proposed to underlie neuronal processing of temporal duration (Wittmann 2013). Neurons in the posterior parietal cortex exhibit this type of ramping activity, which may represent elapsed time before the execution of eye movement (Leon and Shadlen 2003). Ramping activity was also recorded in the motor cortex of monkeys performing self-timed movements (Lebedev et al. 2008), and in the supplementary motor area while monkeys were holding a key during a timed delay period (Mita et al. 2009). These ramping activities peaked at the end of a waiting period and just before the execution of instructed movements. Whether a similar neuronal mechanism for time representation is also found when the stimulus duration is timed for memory and the comparison processes of interval timing are intriguing (Gibbon et al. 1984).

A duration discrimination task is a frequently employed procedure to investigate time perception. In the task, a pair of stimulus durations is presented successively, and the second duration is judged relative to the first. This task requires neuronal processing for parametric scaling, binary encoding of durations, and goal-oriented procedures (Tanji and Mushiake 2009). In previous studies, we recorded neuronal activity from the prefrontal cortex (PFC) while monkeys performed a visual duration discrimination task. PFC neurons show differential activity after the second stimulus presentation whether the second duration is long or short relative to the first (Oshio et al. 2006). Another research group also showed this temporal order-based time representation by PFC neurons (Genovesio et al. 2009). PFC neurons in our study (Oshio et al. 2006) also demonstrated differential activity after the first stimulus presentation according to whether the first duration was long or short relative to the presumed standard duration. Phasic PFC activity during the first presentation with a constant delay after the stimulus onset may serve to categorize the ongoing stimulus (Oshio et al. 2008).

Corticostriatal projections from the PFC terminate rostrally in the striatum (Selemon and Goldman-Rakic 1985; Yeterian and Pandya 1991). To understand the neuronal mechanism underlying interval timing in the cortico-basal ganglia network, examining neuronal activity in the striatum as well as the PFC is necessary. Few studies have so far examined striatal activity in monkeys performing a timing task, although time-stamp of events was represented by striatal and PFC neurons in a sequential saccade task (Jin et al. 2009). In the present study, single-unit activity was recorded from the rostral striatum during the same duration discrimination task as in the previous PFC studies (Oshio et al. 2006, 2008). Because the cortex and basal ganglia are likely to play cooperative roles in interval timing, comparing the striatal activity to that of the PFC will be interesting. The comparison should provide important implications for the functional organization of the cortico-basal ganglia circuit. Preliminary results were presented previously (Chiba et al. 2008).

## Materials and Methods

Two male Japanese monkeys (*Macaca fuscata*, M11 and M16, weighing 7.3 kg and 8.0 kg, respectively) were used in the present study. All experimental protocols were reviewed and approved by the Animal Care and Use Committee of Kinki University and were performed in accordance with the Guidelines for Proper Conduct of Animal Experiments of the Science Council of Japan (2006). The animals were housed in the institutional animal center under daily supervision of veterinary staff. They acclimated themselves to the experimental environment on custom-fitted primate chairs.

### Behavioral task

**Figure 1:**
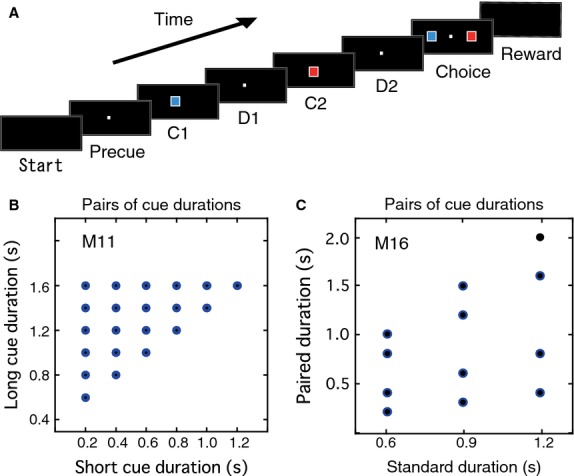
Behavioral task and pairs of cue durations. (A) Duration discrimination task. C1, first cue; D1, first delay; C2, second cue; D2, second delay. (B, C) Pairs of cue durations used in the task for monkeys M11 (B) and M16 (C).

The experimental apparatus and behavioral paradigm were the same as described previously (Oshio et al. 2006, 2008; Chiba et al. 2008). Monkeys sat in a primate chair with their head fixed and faced a flat panel (30 × 40 cm) equipped with a 6.5 inch computer display and three buttons. All visual stimuli were presented on the display against a black background. Each trial (Fig.1A) was initiated when the monkey pressed the center hold button with the right hand, making a white small square (2° × 2°) appear for 1.0 sec at the center of the display. This period was referred to as the precue period. After the precue period, two visual cues (a blue or red square for each, 8° × 8°) of different durations were successively shown at the center of the display. Each of the two cues was followed by a delay period of 1.0 sec in which the small white square (2° × 2°) was presented on the display. The first cue, first delay, second cue, and second delay were referred to as C1, D1, C2, and D2, respectively. After the D2 period, blue and red squares (8° × 8°) were simultaneously presented on the left and right sides on the display, indicating the start of the choice period. If the monkey pressed the target button below the square with the same color as the longer displayed cue within 1.5 sec, the response was judged as correct, and a dispenser squirted a drop of juice into the monkey's mouth 200 msec after the response. No extra feedback signal was given for incorrect responses. The gaze of the monkeys was not controlled during the trial.

Cue duration changed between 0.2 and 2.0 sec, and duration sets for long and short cues overlapped each other. For M11, cue duration varied from 0.2 to 1.6 sec in 0.2 sec increments, with short cues of 0.2–1.2 sec and long cues of 0.6–1.6 sec. A total of 21 long-short duration pairs (Fig.1B) were tested, and the minimum difference between long and short durations was 0.4 sec. With these duration pairs, the monkey could have performed successfully only by comparing the C1 duration with single criterion duration and ignoring C2. To provide lower correct rates with such a simple strategy, we prepared slightly different duration pairs for the other monkey, as shown in our previous paper (Oshio et al. 2008). For M16, cue duration varied from 0.6 to 2.0 sec. Duration pairs were determined as follows; 0.6, 0.9, and 1.2 sec were taken as the standard duration “s”, and variant durations (s/3, 2s/3, 4s/3, 5s/3) were assigned for each standard duration. The resulting 12 duration pairs (Fig.1C) were adopted for this experiment. Thus, a different number of duration pairs was used for each monkey. In each trial, one of the duration pairs was selected for visual cues C1 and C2. The order of relative cue duration was long-short (LS) or short-long (SL), and the cue color sequence was red-blue or blue-red. The duration pair, cue duration order, cue color sequence, and the correct target position (left or right) were pseudorandomly determined. However, the number of duration pairs was limited so that the monkeys could partly predict C2 duration and/or the comparison result after C1 presentation. For example, when the C1 duration was 0.2 sec in M11, the C2 duration was 0.6, 0.8, 1.0, 1.2, 1.4, or 1.6 sec and longer than the C1 duration (Fig.1B). When the C1 duration was 1.0 sec in M16, the C2 duration was 0.6 sec. However, when the C1 duration was 0.9 sec in M16, the C2 duration was 0.3, 0.6, 1.2, or 1.5 sec (Fig.1C). The behavioral paradigm was controlled by personal computers using the TEMPO system (Reflective Computing, St. Louis, MO).

### Single-unit recordings

Single-unit activity was recorded with epoxy-insulated tungsten microelectrodes (1–2.5 MΩ at 1 kHz; FHC, Bowdoinham, ME) from both sides of M11 and the left side of M16 in a dimly lit, electrically shielded room. A microelectrode was advanced into the striatum at an angle of 40° from vertical in the frontal plane through the dura matter with a hydraulic microdrive (Narishige, Japan). The microelectrode was moved in small steps (approximately 5 *μ*m) while monitoring the signal waveform on an oscilloscope and transmitting sound from a speaker. After filtering (band-pass 150–3000 Hz) and amplification, spikes from a single neuron were isolated using a Multi-Spike Detector (MSD, Alpha Omega Engineering, Israel) based on an eight-point template-matching algorithm. If a spike was accepted by the MSD, the accepted signal was also displayed with the corresponding spike on the oscilloscope for confirmation. Visual stimuli and spikes accepted by the MSD were also displayed on the TEMPO display. Judging online whether the activity was related to the task events or not was difficult, partly because durations of the C1 and C2 periods differed from trial to trial. Therefore, the spike data for almost all well-isolated neurons were stored for off-line analysis with millisecond resolution using the TEMPO system. The behavioral performance of the monkeys during the recording was monitored with a CCD camera placed on the ceiling in the shielded room.

### Statistical analysis

Trials with correct responses were submitted for further analysis, although we also believe that analysis of correct versus incorrect trials will be interesting. First, for each neuron, mean firing rates were calculated for the precue, C1, C2, D1, and D2 periods. Mean firing rates during the 1.0 sec precue period were defined as baseline activity. The C1, C2, D1, and D2 activities were compared with the baseline activity using the Mann–Whitney *U*-test (significance level, *P *<* *0.05) in each neuron. When a statistically significant difference was detected in these comparisons, the neuron was defined as a C1, C2, D1, or D2 response neuron. Gaussian fitting analysis of C1 and C2 response neurons was performed to characterize the response by peak time and peak width. Peak width was estimated as “2c”, close to the full width of half maximum, where “c” is the standard deviation of the Gaussian distribution. The details of this analysis were explained in our previous paper (Oshio et al. 2006). In C2 and D1 response neurons, we evaluated changes in activity depending on the C1 duration. Mean firing rates during the C2 and D1 periods were calculated separately for different C1 durations. The C2 and D1 responses were normalized to the maximum C2 and D1 responses, respectively. The relationship between the normalized responses and C1 durations was analyzed using linear regression analysis (significance level, *P *<* *0.05). A gradient and variance of residual were calculated to evaluate dependence of the activity on C1 duration. In D2 response neurons, the activity during the D2 period was compared between LS and SL trials using the Mann–Whitney *U-*test (significance level, *P *<* *0.05). When a statistically significant difference was detected in this comparison, the neuron was defined as a duration-order neuron. Duration-order neurons include LS type neurons that showed greater activity in LS trials than in SL trials, and SL type neurons that showed greater activity in SL trials than in LS trials. We also evaluated changes in D2 activity depending on the C2 duration. Mean firing rates during the D2 period were calculated separately for different C2 durations in LS and SL trials. Using linear regression analysis (significance level, *P *<* *0.05) a gradient and variance of residual were calculated to evaluate dependence of the D2 activity on the C2 duration as the regression analysis of D1 and C2 activity.

We also examined the effects of cue color on the activity. C1 and D1 activity were compared between blue C1 and red C1 trials in C1 and D1 response neurons, respectively, using the Mann–Whitney *U*-test (significance level, *P *<* *0.05). Similarly, C2 activity was compared between blue C2 and red C2 trials in C2 response neurons. When a statistically significant difference was detected in one of these comparisons, the neuron was defined as a color-sensitive neuron. In D2 response neurons, D2 activity was compared between blue-choice and red-choice trials using the Mann–Whitney *U*-test (significance level, *P *<* *0.05) to examine if the activity represented a colored cue that was presented longer, blue or red.

### Histology

At the end of the experiments, to identify the recording sites, electrolytic lesions were placed with passing negative currents (30 *μ*A delivered for 30 sec) through the microelectrode in the striatum. The monkey was deeply anesthetized with an overdose of sodium pentobarbital (Nembutal; 60 mg/kg, i.p.), and perfused transcardially with 0.1 mol/L phosphate-buffered saline (PBS, pH 7.3), followed by 10% formalin dissolved in 0.1 mol/L PBS. The brain was removed immediately from the skull, saturated with 10% then 30% sucrose in 0.1 mol/L PBS at 4°C. The saturated brain was cut serially into 50 *μ*m thick coronal sections on a freezing microtome. Nissl staining was performed in every fourth section to trace the penetrations and verify the electrolytic lesions. Recording sites were reconstructed by reference to marking lesions, and aided by the log notation of the micrometer depths at which different types of neuronal activity were recorded as the electrode was advanced.

## Results

### Correct response rates

**Figure 2:**
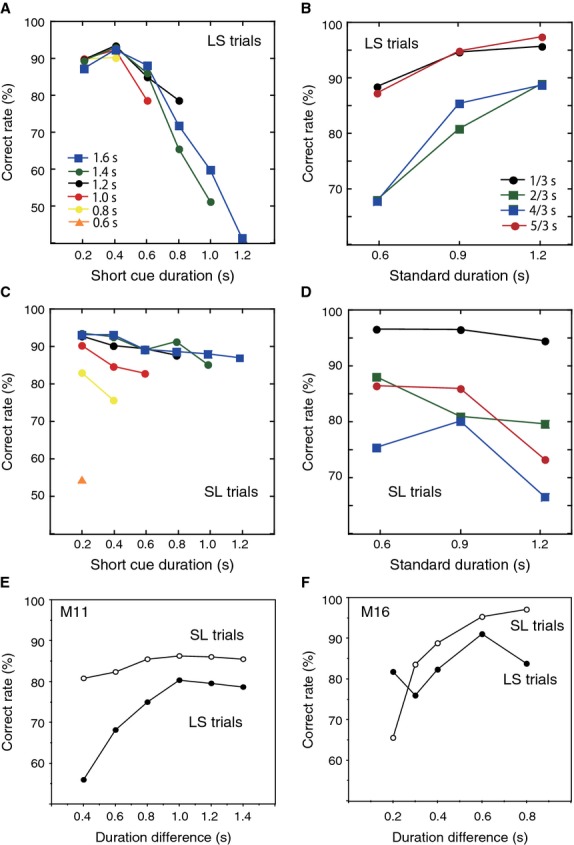
Correct response rates. (A and C) Correct response rates for M11 (24,367 trials) in long-short (LS, (A) and short-long (SL, (C) trials. Correct rates are plotted against short cue durations with the same colored symbols for the same long durations. (B and D) Correct response rates for M16 (23,961 trials) in LS (B) and SL (D) trials. Correct rates are plotted against standard durations using the same colored symbols for the same variant durations. (E and F) Correct rates as a function of duration differences between long and short cues for LS and SL trials in M11 (E) and M16 (F).

For behavioral performance analysis, 24,367 trials were collected from monkey M11 (12260 LS trials and 12107 SL trials) and 23,961 trials from monkey M16 (11952 LS trials and 12009 SL trials) during stable performance sessions in which the monkeys did not take a long rest of more than 1 min and temporal error trials were less than 1% of total trials. A trial was defined as a temporal error when the monkey released the hold button before the go signal or did not press one of the target buttons within 1.5 sec after the go signal. We excluded the temporal error trials from the following behavioral analysis. Percentages of correct responses were calculated separately in LS and SL trials for each of the 21 and 12 duration pairs performed by M11 and M16, respectively. Both monkeys generally performed well in trials with large duration differences and did worse in those with small differences. For example, in LS trails of M11 (Fig.2A), correct rates decreased with accompanying increases in short duration for the same long durations. In LS trials of M16 (Fig.2B), correct rates were higher in trials with variant durations s/3 and 5s/3 than in those with 2s/3 and 4s/3 sec for same standard durations.

However, the monkeys performed differently between LS and SL trials. In SL trials of M11 (Fig.2C), correct rates did not change as dramatically when short durations changed and long durations were constant as was found in LS trials. Instead, correct rates decreased when long duration decreases and short durations were constant. For example, when the short duration was 0.2 sec in SL trials, correct rates decreased as the long duration decreased from 1.6 to 0.6 sec, as shown in the left part of the graph in Figure2C. Some cue duration pairs induced obviously different correct rates between LS and SL trials. For example, correct rates for a pair of 1.6 sec and 1.2 sec were 41% for LS trials and 88% for SL trials in M11 (Fig.2A and C). Weber's law states that a just noticeable difference represents a constant ratio of the absolute strength of stimuli. If Weber's law applied to the performance of M16, the correct rates for each variant duration (s/3, 2s/3, 4s/3, and 5s/3) would have been constant, because the ratios between the two durations of each pair were constant. However, the correct rates for the same variant durations differed between LS and SL trials in M16 except for s/3 in SL trials (Fig.2B and D) with one-way ANOVAs (*P *<* *0.05). Figure2E and F separately demonstrate correct rates as a function of duration differences between long and short cues for LS and SL trials in M11 and M16. Correct rates for the same duration differences differed between LS and SL trials in both monkeys. If two durations had been estimated independently, correct rates would have been similar between LS and SL trials with same variant durations or would have changed similarly according to the duration differences in both trials. However, the monkeys behaved differently than this assumption. These results suggest that C1 and C2 durations were not estimated independently and that encoding of the C1 duration affected the following estimation of the C2 duration in both monkeys.

### Neuronal activity

A total of 498 phasically active neurons were recorded from both sides of the striatum in M11 (*n* = 255) and from the left side in M16 (*n* = 243). Phasically active neurons were defined according to Kimura et al. (1996). Out of the 498 neurons, 269 neurons were defined as task-related neurons, including C1, D1, C2, and D2 response neurons. The numbers of each type of neuron are summarized in Table1, separately from the left and right sides of M11 and the left side of M16. Each type of response neuron was distributed similarly in both hemispheres. In the following paragraphs we describe the activity features of each type of neuron.

**Table 1:** Numbers of responsive neurons during each task period.

	Total	C1	D1	C2	D2
M11 (Left)	55	3	16	17	34
M11 (Right)	109	2	13	40	96
M16 (Left)	105	9	20	37	76
Total^1^	269 (100%)	14 (5%)	49 (18%)	94 (35%)	206 (70%)

1 Some neurons responded during more than one task period.

### C1 response neurons

**Figure 3:**
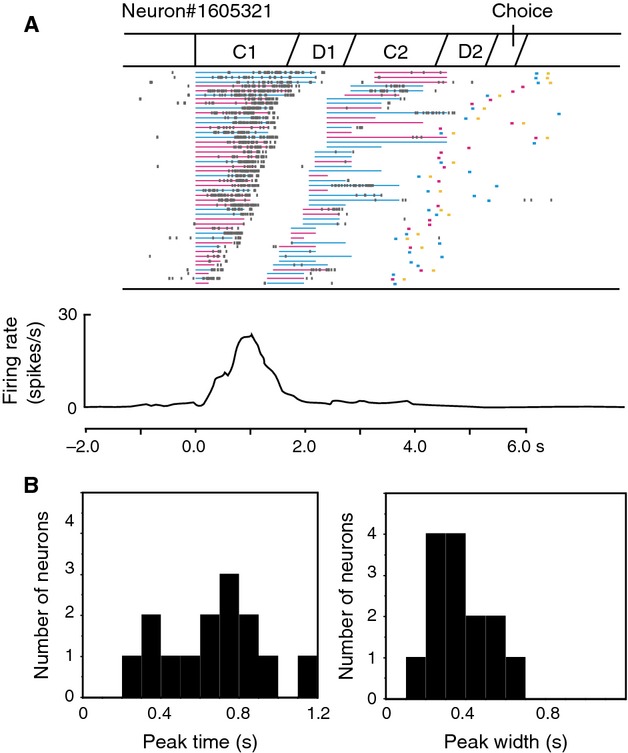
Activity of C1 response neurons. (A) An example of a C1 response neuron. The raster and firing rate curve are aligned with the C1 onset. In the raster display, which is rearranged by the C1 duration from long to short, a row corresponds to a trial. In each row, small black dots represent times of neuronal firing, and red and blue horizontal lines denote periods of colored cue presentation. Red and blue points correspond to times and colors of the subject's choice. Yellow points represent times of reward delivery. Firing rates were averaged in sliding time windows of 200 msec, moving in 20 msec steps. (B) Distributions of peak times from the C1 onset (left) and peak widths (right) of C1 activity for all C1 response neurons (*n* = 14).

Striatal neurons were not as active during C1 presentations as in other task periods. Comparatively few neurons (*n* = 14) were defined as C1 responsive, and only two neurons of these were color sensitive. Figure3A shows an example of a C1 response neuron. This neuron exhibited phasic activity during the C1 period with a peak time of 0.8 sec from the C1 onset and a peak width of 0.5 sec according to the Gaussian fitting analysis. Distributions of the peak time and peak width for all C1 response neurons are summarized in Figure3B. The peak time was unimodally distributed with a median of 0.7 sec. The peak width ranged mainly around 0.2–0.6 sec with a median of 0.4 sec, and sustained activity throughout the C1 presentation was not found. The same analysis was performed for PFC activity (Oshio et al. 2008). Peak times and peak widths were similarly distributed in striatal and PFC activity, although the number of C1 response neurons was quite small in the striatum.

### D1 response neurons

**Figure 4:**
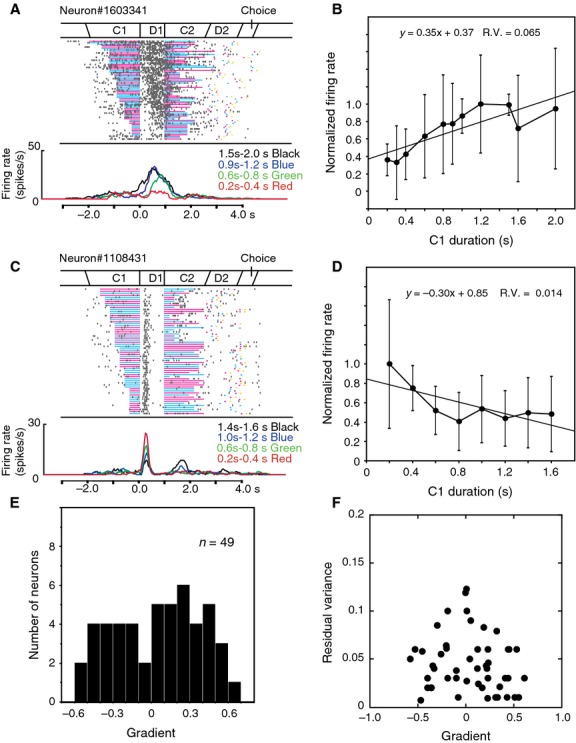
Activity of D1 response neurons. (A) An example of a D1 response neuron exhibiting greater D1 activity following long C1s. The raster is aligned with the onset of the D1 period and rearranged by the C1 duration. Firing rate curves are separately presented for four groups of C1 durations (0.2–0.4, 0.6–0.8, 0.9–1.2, and 1.5–2.0 sec) with different colors under the raster display. (B) Relationship between D1 activity and the C1 duration for the neuron shown in panel A. Normalized firing rates during the D1 period are plotted against the C1 duration as mean ± standard deviation. Linear regression analysis revealed a positive gradient (0.35) with a small residual variance (0.065). (C) An example of a D1 response neuron exhibiting greater D1 activity following short C1s. Firing rate curves are separately presented for four groups of C1 durations (0.2–0.4, 0.6–0.8, 1.0–1.2, and 1.4–1.6 sec). (D) Relationship between D1 activity and the C1 duration for the neuron shown in panel C. Linear regression analysis revealed a negative gradient (−0.30) with a small residual variance (0.014). (E) Distribution of the gradient from the linear regression analysis of D1 activity for 49 D1 response neurons. (F) A scatter plot of the gradient and residual variance from the linear regression analysis in 49 D1 response neurons.

Nearly one-fifth of task-related neurons (*n* = 49) were defined as D1 responsive, and 21 neurons of these were color sensitive. Some D1 response neurons changed their activity depending on the C1 duration. Figure4 shows two examples of these neurons. The neuron shown in Figure4A exhibited phasic activity during the D1 period. The activity was higher in trials with a long C1 than in those with a short one. The dependence of D1 activity on the C1 duration was quantitatively evaluated for this neuron. Mean firing rates during the D1 period were calculated separately for different C1 durations. The mean D1 responses were normalized to the maximum D1 response. The relationship between normalized firing rates and C1 durations was examined with linear regression analysis, which revealed the dependence of the D1 activity on the C1 duration with a positive gradient (a = 0.35, *P *<* *0.001, Fig.4B). A longer C1 duration induced greater D1 activity. A small residual variance (0.065) ensured reliable linearity.

The neuron shown in Figure4C exhibited phasic D1 activity, which was higher following a short C1 than after a long one. The relationship between normalized firing rates and C1 durations was examined with linear regression analysis, which revealed the dependence of the D1 activity on the C1 duration with a negative gradient (a = −0.30, *P *<* *0.001, Fig.4D). A shorter C1 duration induced higher D1 activity. A small residual variance (0.016) ensured reliable linearity.

The same linear regression analysis was performed on 49 D1 response neurons. Figure4E shows the distribution of the gradients of the activity revealed by this analysis. Some of the neurons exhibited the activity with positive or negative trends. Based on the gradient “a” determined with this analysis, D1 activity was divided into three patterns, positive (a > 0.3), gradual (0.3 >  a  >  −0.3), and negative (−0.3 >  a). The numbers of D1 response neurons with each pattern are summarized in Table2. The gradients and residual variances for each neuron are plotted in Figure4F. Neurons with greater absolute values of the gradient tended to have smaller residual variances, indicating that neurons with positive and negative trends showed a reliable linear relationship between the C1 duration and D1 activity. Striatal neurons may represent the C1 duration with monotonically changing activity during the D1 period after scaling the stimulus duration in the C1 period.

**Table 2:** Numbers of D1- and D2-response neurons with positive, gradual, and negative patterns.

	Total	Positive	Gradual	Negative
D1	49 (100%)	13 (27%)	26 (53%)	10 (20%)
D2				
LS-type	112 (100%)	18 (16%)	50 (45%)	44 (39%)
SL-type	33 (100%)	4 (12%)	14 (42%)	15 (46%)
Non^1^	61	–	–	–

1 D2 response neurons that did not exhibited significantly different D2 activity between LS and SL trials are classified as “Non”.

### C2 response neurons

**Figure 5:**
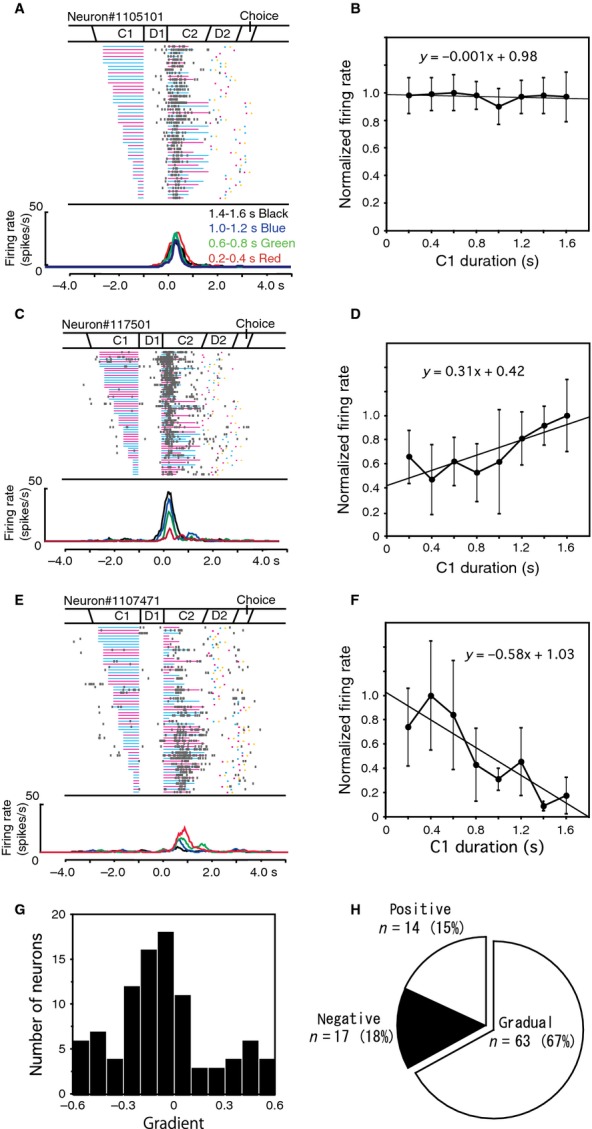
Activity of C2 response neurons. (A) An example of a C1-independent type of C2 response neuron. The raster is aligned with the C2 onset and rearranged by the C1 duration. Firing rate curves are presented separately for four groups of C1 durations. (B) Normalized firing rates during the C2 period are plotted against the C1 duration in the neuron shown in panel A. (C) An example of a positive type of C2 response neuron. (D) Normalized firing rates during the C2 period are plotted against C1 duration for the neuron shown in panel C. Linear regression analysis revealed a positive gradient (0.31). (E) An example of a negative type of C2 response neuron. (F) Normalized firing rates during the C2 period are plotted against C1 durations for the neuron shown in panel E. Linear regression analysis revealed a negative gradient (−0.58). (G) Distribution of the gradient from the linear regression analysis of the C2 activity of 94 C2 response neurons. (H) Numbers of each type of C2 response neuron.

Almost one-third of the task-related neurons (*n* = 94) were defined as C2 responsive, and 24 of these were color sensitive. Some of C2 response neurons changed their activity depending on the C1 duration. Figure5 shows three examples of C2 response neurons. The neuron shown in Figure5A exhibited phasic activity during the C2 period with an early peak time of 303 msec from the C2 onset. Mean firing rates during the C2 period were calculated separately for different C1 durations and normalized to the maximum C2 response. The relationship between normalized firing rates and C1 durations was examined with linear regression analysis, which revealed no dependence of the activity on the C1 duration (Fig.5B). On the other hand, the neuron shown in Figure5C exhibited phasic C2 activity, which changed depending on the C1 duration. A longer C1 duration induced greater C2 activity. Linear regression analysis revealed the dependence of the C2 activity on the C1 duration with a positive gradient (a = 0.31, *P *=* *0.03, Fig.5D). The neuron shown in Figure5E exhibited phasic activity with a late peak time of 735 ms from the C2 onset. This activity also changed depending on the C1 duration, although a shorter C1 duration induced greater C2 activity. Linear regression analysis revealed the dependence of the C2 activity on the C1 duration with a negative gradient (a = −0.58, *P *=* *0.005, Fig.5F). The same linear regression analysis was performed on 94 C2 response neurons. Figure5G shows the distribution of the gradients of these neurons as revealed by the analysis. Based on the gradient “a” determined from this analysis, C2 activity was divided into three patterns, positive (a > 0.3), gradual (0.3 > a > −0.3), and negative (−0.3 > a) types. The numbers of C2 response neurons with each pattern are summarized in Figure5H. Positive and negative types of neurons may represent C1 durations with time-varying signals using similar strategies as D1 activity.

**Figure 6:**
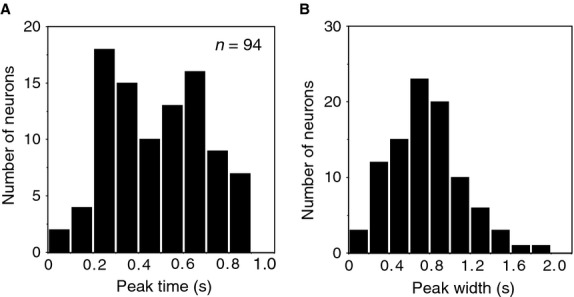
Distributions of peak times from the C2 onset (A) and peak widths (B) of C2 activity in 94 C2 response neurons.

Peak times and peak widths of 94 C2 response neurons were characterized with the Gaussian fitting analysis. Distributions of the peak time and peak width are summarized in Figure6. Distribution of the peak times showed the main peak at 0.3 sec and the second one around 0.7 sec from the C2 onset (Fig.6A). The peak widths ranged mainly around 0.2–1.0 sec. (Fig.6B). Activity with long latency may contribute to detection of a longer C2 duration. Investigating the relationship between C2 activity and the C2 duration will be worthwhile. However, most of C2 response neurons exhibited early phasic responses after the C2 onset (Fig.6A), and we are unsure of how to analyze this relationship.

### D2 response neurons

**Figure 7:**
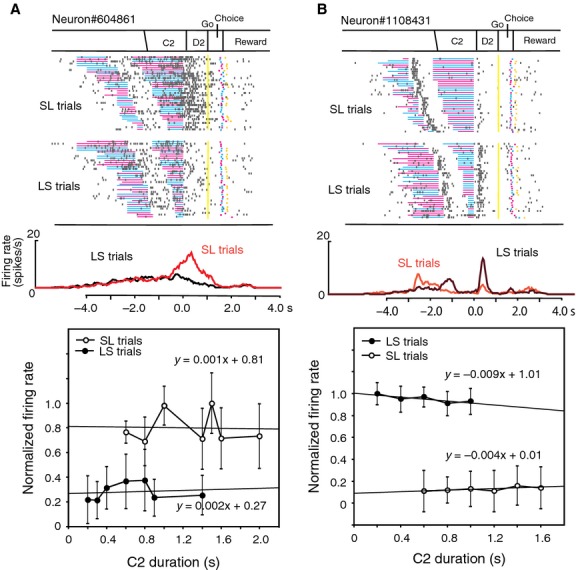
D2 response neurons representing the order of relative cue duration. (A) An example of an SL type of D2 response neuron. *Top*; The raster diagram, which is aligned with the D2 onset and rearranged by the C2 duration, is displayed separately for SL and LS trials. *Middle;* Firing rate curves are shown separately for SL (red) and LS (black) trials. *Bottom;* Normalized firing rates are plotted separately for SL and LS trials against the C2 duration. (B) An example of an LS type of D2 response neuron.

Nearly 70% of task-related neurons (*n* = 206) were defined as D2 responsive. Two thirds of D2 response neurons (*n* = 145) showed differential activity during the D2 period between LS and SL trials, representing the order of relative cue duration (long-short or short-long). Figure7 shows two examples of such duration-order D2 response neurons. The neuron shown in Figure7A exhibited significantly greater D2 activity in SL trials than in LS trials. This type of neuron is defined as SL type. On the other hand, the neuron in Figure7B exhibited greater D2 activity in LS trials than in SL trials, and is defined as LS type. The activity of these neurons changed according to the trial type, but did not change within the same type of trials even if the C2 duration was altered.

**Figure 8:**
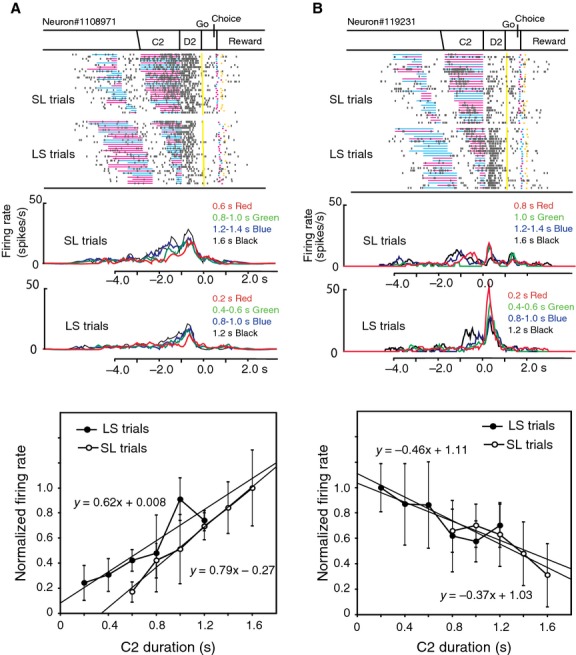
D2 response neurons exhibiting C2 duration-dependent activity. (A) An example of a D2 response neuron with increased D2 activity with elongation of the C2 duration. *Top;* The raster diagram, which is aligned with the D2 onset and rearranged by the C2 duration, is displayed separately for SL and LS trials. *Middle;* Firing rate curves for each C2 duration group are shown with different colors, and are shown separately for SL and LS trials. The colored curves represent firing rates for four duration groups for clear presentation, although the firing rates for each duration were used in the following linear regression analysis. *Bottom,* Normalized firing rates are plotted against the C2 duration, and are shown separately for SL and LS trials. Linear regression analysis revealed similar positive gradients for SL (0.79) and LS trials (0.62). (B) An example of a D2 response neuron with decreased D2 activity with elongation of the C2 duration. Linear regression analysis revealed similar negative gradients for SL (−0.37) and LS trials (−0.46).

Some D2 response neurons changed their activity depending on the C2 duration. Figure8 demonstrates two examples of neurons exhibiting C2-dependent D2 activity. The neuron shown in Figure8A exhibited phasic activity during the D2 period both in SL and LS trials. The magnitude of the D2 activity changed depending on the C2 duration. The activity was greater in trials with a long C2 than in those with a short one. Normalized firing rates during the D2 period were plotted separately for SL and LS trials against the C2 duration (Fig.8A *bottom*). Linear regression analyses revealed the dependence of D2 activity on the C2 duration with a positive gradient both in SL trials (a = 0.79, *P *=* *0.01) and in LS trials (a = 0.62, *P *=* *0.01). The neuron shown in Figure8B also exhibited phasic activity during the D2 period. The activity was greater in trials with a short C2 than in those with a long one. Normalized firing rates during the D2 period were plotted separately for SL and LS trials against the C2 duration (Fig.8B *bottom*). Linear regression analyses revealed the dependence of D2 activity on the C2 duration with a negative gradient both in SL trials (a  =  −0.37, *P *<* *0.001) and in LS trials (a  =  −0.46, *P *<* *0.001). No appreciable difference was found in the gradient between LS and SL trials in these neurons.

**Figure 9:**
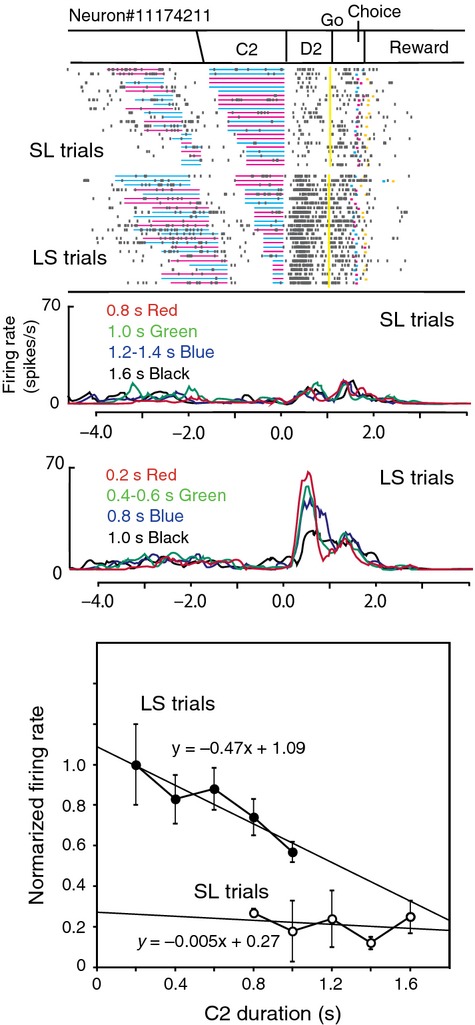
An example of a D2 response neuron with decreased D2 activity with elongation of the C2 duration only in LS trials. *Top;* The raster diagram, which is aligned with the D2 onset and rearranged by the C2 duration, is displayed separately for SL and LS trials. *Middle;* Firing rate curves for four C2 duration groups are shown with different colors and are shown separately for SL and LS trials. *Bottom;* Normalized firing rates are plotted against the C2 duration, separately for SL and LS trials. Linear regression analysis revealed a negative gradient (−0.47) only in LS trials.

On the other hand, the neuron in Figure9 showed greater D2 activity in LS trials than in SL trials, and the D2 activity changed depending on the C2 duration in LS trials, but not in SL trials. Linear regression analyses revealed the dependence of D2 activity on the C2 duration with a negative gradient (−0.47, *P *<* *0.001) in LS trials and but not in SL trials (Fig.9 *bottom*).

**Figure 10:**
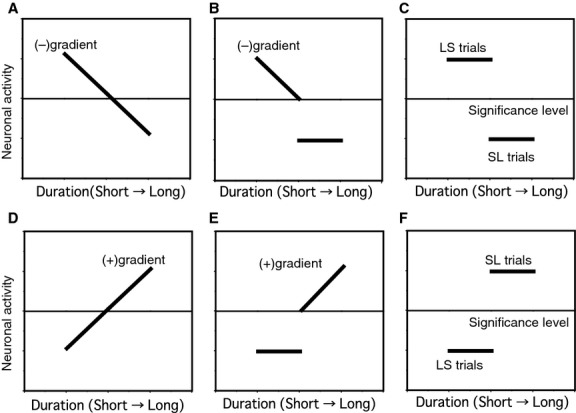
Schematic diagrams showing D2 activity patterns in D2 response neurons based on C2 duration dependence and duration-order types. In each diagram D2 activity is schematically plotted against the C2 duration. The activity is shown separately for LS and SL trials in panels B, C, E, and F. The middle horizontal line in each diagram demonstrates the significance level of the activity set for temporal judgment.

Figure10 illustrates schematic diagrams showing D2 activity patterns in D2 response neurons based on C2 duration dependence and duration-order type. In patterns A and D, the activity changed monotonically depending on the C2 duration. As the C2 duration was lengthened, the activity decreased in pattern A as shown for the neuron in Figure8B, whereas the activity increased in pattern D as shown for the neuron in Figure8A. In patterns C and F, the activity simply represented the duration order. Greater activity was found during LS trials in pattern C as shown for the neuron in Figure7B, whereas greater activity was observed during SL trials in pattern F as shown for the neuron in Figure7A. In patterns B and E, the activity changed depending on the C2 duration only in dominant trials. The activity increased as the C2 duration shortened only in LS trials in pattern B as shown for the neuron in Figure9, whereas the activity increased as the C2 duration lengthened only in SL trials in pattern E. All these types of neurons represent discrimination results between two cue durations when a significant level of activity is set for temporal judgment, in addition to cue duration in patterns A to D. The “significance level” is arbitrary in this figure, and we did not find a neuronal mechanism with which to compute this level. From the recorded neuronal activity, we found these patterns of D2 activity and classified D2 response neurons based on differences in firing rates and gradients from linear regression analysis between LS and SL trials. The numbers of each pattern of neurons are summarized in Table3.

**Table 3:** Numbers of D2-response neurons with patterns A to F as shown in Figure9,

Total	A	B	C	D	E	F
145 (100%)	17 (12%)	36 (25%)	59 (40%)	3 (2%)	0 (0%)	30 (21%)

**Figure 11:**
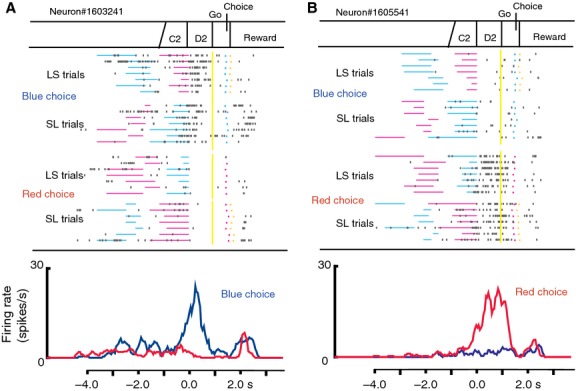
D2 response neurons representing colors of relative cue durations. (A) An example of a D2 response neuron with greater D2 activity in blue-choice trials. The raster and firing rate curves are displayed separately for blue- and red-choice trials and are aligned with the D2 onset. (B) An example of a D2 response neuron with greater D2 activity in red-choice trials.

Some D2 response neurons showed differential activity according to which colored cue, blue or red, was presented longer. Figure11 demonstrates two examples of such D2 response neurons. The neuron shown in Figure11A showed greater D2 activity when the blue cue was presented longer irrespective of the presenting order, in LS or SL trials. On the other hand, the neuron shown in Figure11B exhibited greater D2 activity when the red cue was presented longer. These activities are similar to the PFC activity that represents feature-based timing reported by Genovesio et al. (2009). Twenty-three neurons (12 neurons in M11 and 11 in M16) showed this type of activity representing color-based timing.

### Recording sites

Recording sites were reconstructed by reference to marking lesions, aided by the log notation of the depths at which different types of neuronal activity were recorded as the electrode was advanced. Recorded neurons were mainly distributed in the rostral portion of the caudate nucleus and the rostromedial part of the bilateral putamen, from A26 to A18 according to the brain atlas of *Macaca fuscata* (Mabuchi and Kusama 1970). Precise recording sites in M11 were shown in our previous paper (Chiba et al. 2008), and M16 has not been sacrificed. Each type of response neurons was widely found in these regions.

## Discussion

In the present study, we investigated neuronal activity of the monkey striatum during a duration discrimination task to clarify the functional roles of the basal ganglia in interval timing. We found several types of task-related activity in the striatum. Among them, two types of activity during the second cue, and/or the first and second delay periods were quite interesting. The first type was the activity that gradually changed depending on the duration of the cue presented just before. This activity may encode the signal duration for later comparison between two cue durations. The second type was the activity that differentially changed whether the first or second cue was presented longer. This activity may encode discrimination results after a comparison between two signal durations.

### Neuronal responses

The present findings support the idea that striatal neurons encode timing information of sensory signals for duration discrimination. Two types of the striatal activity are possibly involved in timing representation. First, striatal neurons changed their activity depending on the C1 duration during the D1 and C2 periods. Some neurons increased their activity as the C1 duration was lengthened, whereas other neurons did so as the C1 duration was shortened. These activities may represent duration of the sensory signals after signal duration was parametrically scaled for later cognitive processes. The neuronal mechanism for scaling of the C1 duration and induction of parametric responses to the timed duration has not been elucidated, although striatal neurons may integrate pulses in a linear manner during cue presentation. Only our previous report (Chiba et al. 2008) has described that the magnitude of neuronal activity in the striatum represents the signal duration. D1 activity may reflect anticipation of the C2 duration or long/short categorization of the C1 duration because the C2 duration and/or the categorization result may be partially predictable after C1 presentation.

Second, striatal neurons demonstrated differential activity between LS and SL trials during the D2 period after the two cue presentations. In LS trials C1 was presented longer than C2, and C2 was presented longer in SL trials. Therefore, this activity may represent the relative duration of the two cues, that is, which was presented longer. This order-based timing representation should lead to the final binary decision for the correct choice in the duration discrimination task. Early parametric information regarding the signal duration may be transformed to the later binary information for the final decision in the striatum or through the cortico-basal ganglia loop circuit. In the PFC, the same type of binary activity representing order-based timing has been found in the same task (Oshio et al. 2006; Genovesio et al. 2009). Although the functional roles of the PFC and the basal ganglia have not been clarified, these two areas have close anatomical interconnections and may cooperate to discriminate temporal duration of sensory signals.

Multiple ways may be used to represent time information in striatal neurons. In duration discrimination, the neural system may adopt different ways to represent the first and second sensory signals. Furthermore, interval timing should be implemented over multiple brain regions in an overlapping manner (Merchant et al. 2013; Wittmann 2013). Many different firing patterns were found in the present study. Examining whether similar activity patterns are present in the static condition in which one of the durations is constant as a control will be interesting.

In the posterior parietal cortex (Leon and Shadlen 2003), the medial premotor cortex (Mita et al. 2009; Merchant et al. 2011), and the motor thalamus (Tanaka 2007) of the monkey, the elapsed time appears to be represented in buildup of neuronal activity. However, building up of neuronal activity was not found during the presentation of timed signals in the present experiment. The difference in the time course of the activity between these studies and the present experiment may be due to assigned tasks requiring interval timing, motor control, or sensory cognition. Neural integration may produce differences in neuronal activity to be utilized as cues for the required duration depending on the context.

### Behavioral performances

Duration discrimination requires several components of information processing, including timing of signal duration, working memory of the timed duration, and comparison between two signal durations (Gibbon et al. 1984; Meck 1996). The temporal order of two signals influences the final comparative decision. In the present study, task performance was affected by the order of short and long cues as well as by the difference between the two cue durations. In LS trials, correct response rates decreased as the short cue duration lengthened with the long cue duration constant (Fig.2A), whereas correct rates were relatively constant regardless of changes in the short cue duration in SL trials (Fig.2C). Similar differences in correct rates were observed between LS and SL trials in the other monkey (Fig.2B and D). In the present study, the number of duration pairs was different between the two monkeys. Although this difference may affect behavior patterns, a similar order effect was observed in both monkeys.

A couple of assumptions may explain this order effect. Accumulated activity representing the C1 duration may deteriorate and decrease during the memory process. Due to this decrease in activity, C1 duration would be perceived as shorter than it really was. If a longer C1 was perceived as shorter, correct rates would decrease in LS trials, but not in SL trials. During the timing process of C2, more activity may accumulate than during that of C1 because of an attentive or motivated state. Alternatively, more information processing may progress during C2 presentation if the subjects have encoded the C2 duration while comparing it with the C1 duration. Due to these increases in neuronal processing, the C2 duration would be perceived as longer than it really was. If the shorter C2 was perceived as longer, correct rates would also decrease in LS trials, but not in SL trials. In SL trials, differences between two cue durations or the ratio of two durations mainly determined the discrimination accuracy. Smaller differences in duration induced lower correct response rates.

### Roles of the cortico-basal ganglia loop in timing function

Although the present results demonstrate the possibility that activity of striatal neurons represents temporal information of sensory signals, other brain regions are also closely involved in interval timing for duration discrimination. Neuronal activity in the PFC, which provides a major centrifugal output to the striatum, represents order-based timing information in the duration discrimination task (Oshio et al. 2006; Genovesio et al. 2009), as the striatal activity did in the present study. Although functional differences in temporal discrimination are indefinite between the PFC and striatum, the phasic activity with constant latency after cue onset, which could be utilized for temporal filtering of signals, was only found in the PFC (Oshio et al. 2008), but not in the striatum.

A number of models have been proposed to describe how the brain perceives passage of time. These models can be classified into two general frameworks (Ivry and Schlerf 2008). One type is the intrinsic model in which the sensory and cognitive systems, which are not specialized for interval timing, automatically measure signal duration. For example, in the state-dependent network model cortical networks can inherently tell time as a result of time-dependent changes in the network state (Karmarkar and Buonomano 2007). The other type is the dedicated model in which specialized neural systems operate for measuring duration. One of these models is the striatal beat-frequency model (Matell and Meck 2004; Buhusi and Meck 2005). In this model timing is based on coincidental activation of striatal neurons by oscillatory activity of cortical neurons. The cortical oscillatory activities are synchronized at the onset of an event, and striatal neurons detect the specific pattern of oscillatory activity after the interval to be measured. Although we have recorded cortical and striatal activity during the same duration discrimination task, simultaneous multiple recordings from these areas are desirable to validate this model.

Functional brain imaging studies in humans and lesion experiments in animals have indicated that the cortical-basal ganglia circuit is the core structure involved in time perception (Merchant et al. 2013). Many imaging studies have shown that the basal ganglia and the supplementary motor area are routinely engaged during interval timing (Coull et al. 2011). One of these studies separately demonstrated neural activation associated with encoding, maintenance, and decision periods of the time perception task, and showed that striatal activation is specific to interval encoding (Harrington et al. 2010). On the other hand, injection of a dopamine D2 antagonist induced overestimation of intervals in the peak-interval procedure in rats (Drew et al. 2003). Rats that received 6-hydroxydopamine microinjections into the caudate-putamen were unable to maintain temporal control in this procedure (Meck 2006a,b). These results suggest that the nigrostriatal dopaminergic system may affect striatal activity and modulate clock-speed in temporal cognition.

To summarize, striatal neurons encoded signal duration in the duration discrimination task. The first cue duration was represented by gradually changing activity according to the signal duration. Relative duration of the first and second cues was represented by binary changes in activity after presentation of the two signals. This type of activity was found in the prefrontal cortex during the same task (Oshio et al. 2006). The basal ganglia and the PFC likely cooperate to discriminate temporal duration of visual signals.

## References

[b1] Allman MJ, Meck WH (2012). Pathophysiological distortions in time perception and timed performance. Brain.

[b2] Buhusi CV, Meck WH (2005). What makes us tick? Functional and neural mechanisms of interval timing. Nat. Rev. Neurosci.

[b3] Chiba A, Oshio K, Inase M (2008). Striatal neurons encoded temporal information in duration discrimination task. Exp. Brain Res.

[b4] Coull JT, Cheng R-K, Meck WH (2011). Neuroanatomical and neurochemical substrates of timing. Neuropsychopharmacol.

[b5] Drew MR, Fairhurst S, Malapani C, Horvitz JC, Balsam PD (2003). Effects of dopamine antagonists on the timing of two intervals. Pharmacol. Biochem. Behav.

[b6] Genovesio A, Tsujimoto S, Wise SP (2009). Feature- and order-based timing representations in the frontal cortex. Neuron.

[b7] Gibbon J, Church RM, Meck WH (1984). Scalar timing in memory. Ann. NY. Acad. Sci.

[b8] Harrington DL, Zimbelman JL, Hinton SC, Rao SM (2010). Neural modulation of temporal encoding, maintenance, and decision processes. Cereb. Cortex.

[b9] Ivry RB, Schlerf JE (2008). Dedicated and intrinsic models of time perception. Trends Cogn. Sci.

[b10] Ivry RB, Spencer RMC (2004). The neural representation of time. Curr. Opin. Neurobiol.

[b11] Jin DZ, Fujii N, Graybiel AM (2009). Neural representation of time in cortico-basal ganglia circuits. Proc. Natl Acad. Sci. USA.

[b12] Karmarkar UR, Buonomano DV (2007). Timing in the absence of clocks. Neuron.

[b13] Kimura M, Kato M, Shimazaki H, Watanabe K, Matsumoto N (1996). Transferred from the putamen to the globus pallidus during learned movement in the monkey. J. Neurophysiol.

[b14] Lebedev MA, O'Doherty JE, Nicolelis MA (2008). Decoding of temporal intervals from cortical ensemble activity. J. Neurophysiol.

[b15] Leon MI, Shadlen MN (2003). Representation of time by neurons in the posterior parietal cortex of the macaque. Neuron.

[b16] Mabuchi M, Kusama T (1970). Mesodiencephalic projections to the inferior olive and the vestibular and perihypoglossal nuclei. Brain Res.

[b17] Matell MS, Meck MH (2004). Cortico-striatal circuits and interval timing: coincidence detection of oscillatory processes. Cogn. Brain Res.

[b18] Meck WH (1996). Neuropharmacology of timing and time perception. Cog. Brain Res.

[b19] Meck WH (2006a). Frontal cortex lesions eliminate the clock speed effect of dopaminergic drugs on interval timing. Brain Res.

[b20] Meck WH (2006b). Neuroanatomical localization of an internal clock: A functional link between mesolimbic, nigrostriatal, and mesocortical dopaminergic systems. Brain Res.

[b21] Merchant H, Zarco W, Perez O, Prado L, Bartolo R (2011). Measuring time with different neural chronometers during a synchronization-continuation task. Proc. Natl Acad. Sci. USA.

[b22] Merchant H, Harrington DL, Meck WH (2013). Neural basis of the perception and estimation of time. Ann. Rev. Neurosci.

[b23] Mita A, Mushiake H, Shima K, Matsuzaka Y, Tanji J (2009). Interval time coding by neurons in the presupplementary motor areas. Nat. Neurosci.

[b24] Oshio K, Chiba A, Inase M (2006). Delay period activity of monkey prefrontal neurons during duration-discrimination task. Eur. J. Neurosci.

[b25] Oshio K, Chiba A, Inase M (2008). Temporal filtering by prefrontal neurons in duration discrimination. Eur. J. Neurosci.

[b26] Selemon LD, Goldman-Rakic PS (1985). Longitudinal topography and interdigitation of corticostriatal projections in the rhesus monkey. J. Neurosci.

[b27] Tanaka M (2007). Cognitive signals in the primate motor thalamus predict saccade timing. J. Neurosci.

[b28] Tanji J, Mushiake H (2009). Which object appeared longer?. Neuron.

[b29] Teki S, Grube M, Griffiths TD (2012). A unified model of time perception accounts for duration-based and beat-based timing mechanisms. Front. Integr. Neurosci.

[b30] Wittmann M (2013). The inner sense of time: how the brain creates a representation of duration. Nat. Rev. Neurosci.

[b31] Yeterian EH, Pandya DN (1991). Prefrontostriatal connections in relation to cortical architectonic organization in rhesus monkeys. J. Comp. Neurol.

